# Risk factors for infection after endoscopic ultrasonography-guided drainage of specific types of pancreatic and peripancreatic fluid collections (with video)

**DOI:** 10.1007/s00464-015-4557-3

**Published:** 2016-01-22

**Authors:** Jintao Guo, Linlin Feng, Siyu Sun, Nan Ge, Xiang Liu, Sheng Wang, Guoxin Wang, Beibei Sun

**Affiliations:** Endoscopy Center, Shengjing Hospital of China Medical University, No. 36, Sanhao Street, Shenyang, 110004 Liaoning Province China

**Keywords:** EUS-guided drainage, Peripancreatic fluid collections, Infection, Complications

## Abstract

**Background:**

Endoscopic ultrasonography (EUS)-guided drainage is widely used for the treatment of specific types of peripancreatic fluid collections (PFCs). Infectious complications have been reported. It is recommended that the infection rate should be assessed by measuring risk factors. The objectives of this study were to measure whether the risk of infection after EUS-guided drainage was associated with patient- and procedure-related factors.

**Methods:**

Eighty-three patients were eligible for inclusion from September 2008 to November 2012. EUS-guided drainage was performed in all patients. Infectious complications were observed, and data on patient- and procedure-related factors were collected. Patient-related factors mainly included age, sex, etiology of PFC, and cyst location and diameter. Procedure-related factors mainly included approach of EUS-guided drainage and stent diameter. Separate multivariate logistic regression models for all EUS-guided drainage were carried out.

**Results:**

Complete EUS-guided drainage was achieved in all patients. A definitive diagnosis of infection after EUS-guided drainage was made in seven patients. All seven patients had a history of acute pancreatitis, and the cyst diameters were all >15 cm. Three patients had diabetes mellitus.

**Conclusions:**

The cyst diameter was an independent risk factor for infection. Larger cysts with a diameter >15 cm should perhaps be drained initially with multiple pigtail or a larger diameter self-expandable metal stents to try to avoid infection.

**Electronic supplementary material:**

The online version of this article (doi:10.1007/s00464-015-4557-3) contains supplementary material, which is available to authorized users.

PFC may complicate the course of pancreatitis, pancreatic surgery, or trauma. Several treatment options are available including surgery, external percutaneous drainage, and internal endoscopic drainage. EUS-guided drainage of PFC is a minimally invasive procedure and has become standard therapy worldwide for pancreatic pseudocyst and pancreatic walled-off necrosis [1, 2].

Infection is one of the common complications after EUS-guided drainage, and it can prolong disease duration and increase length of hospital stay. The fever caused by infection can cause an imbalance in energy consumption and water and electrolyte balance. In the present study, we focused on the risk of infection after EUS-guided drainage. Our objectives were to measure whether the risk of infection after EUS-guided drainage was associated with patient- and procedure-related factors.

## Materials and methods

### Patients

**Fig. 1:**
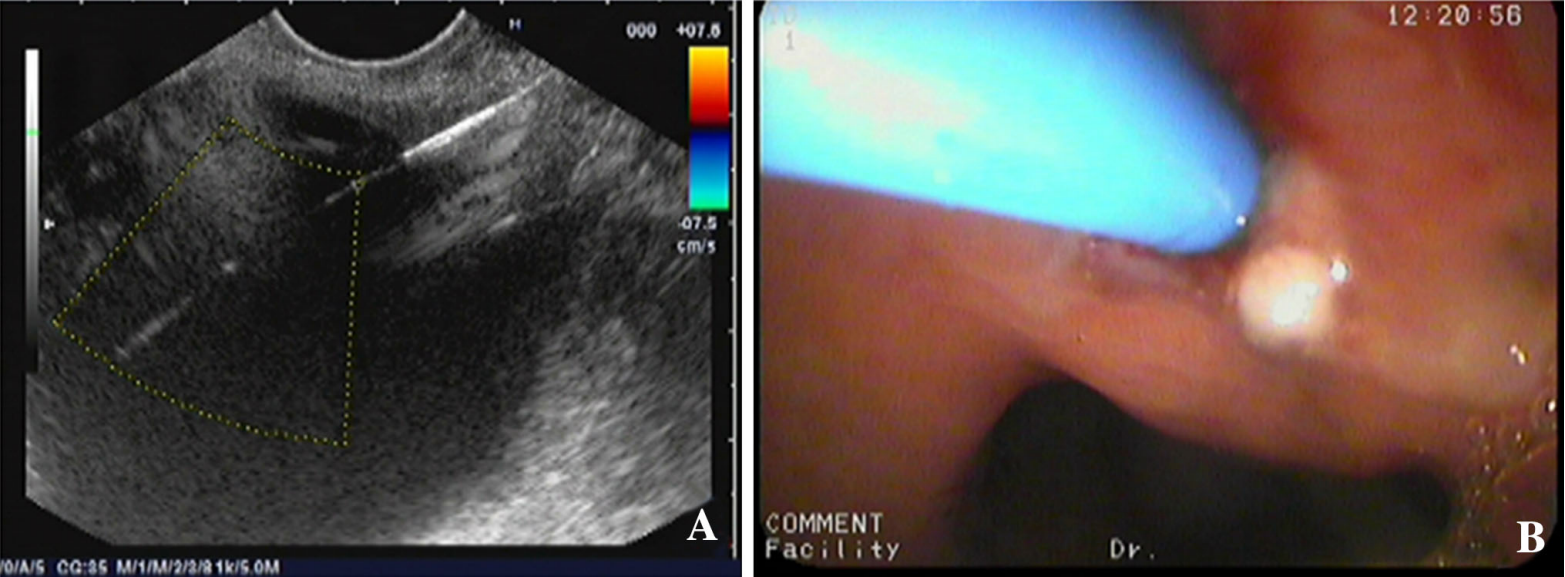
**A** Pancreatic pseudocyst was observed by EUS. **B** EUS-guided drainage was performed transgastrically

**Fig. 2:**
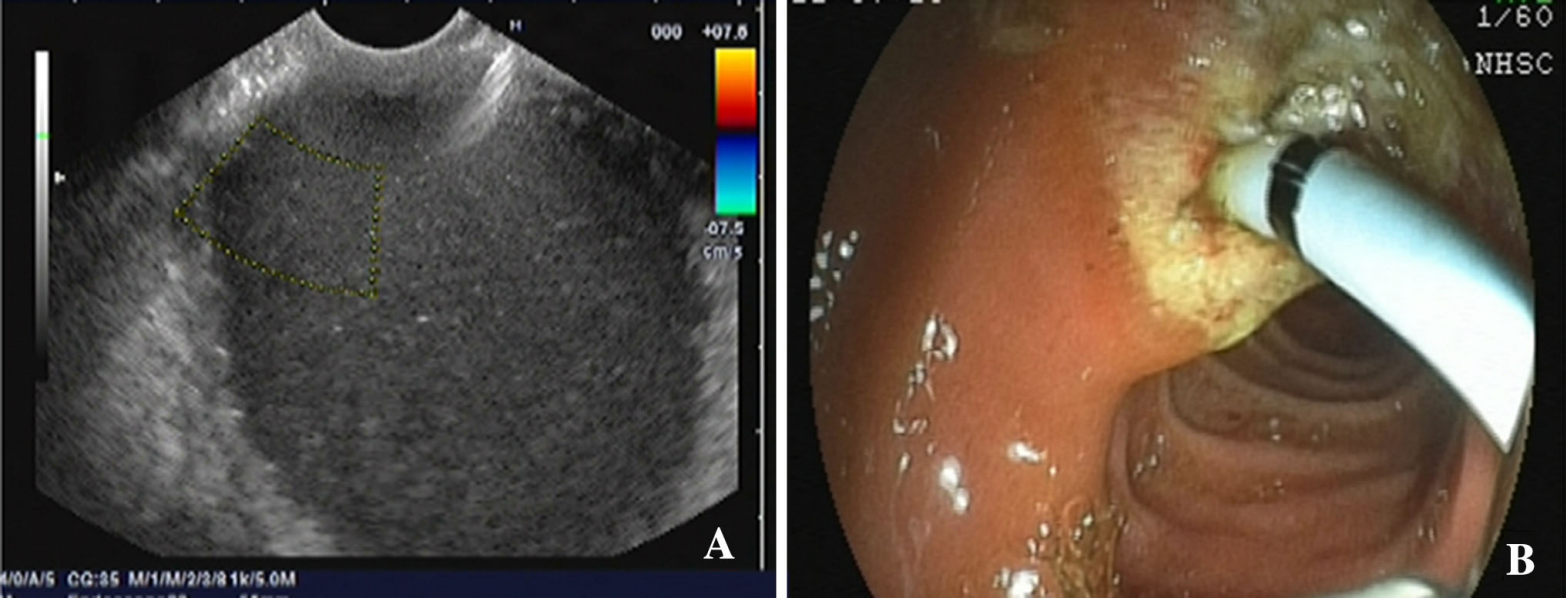
**A** Pancreatic pseudocyst was observed by EUS. **B** EUS-guided drainage was performed transduodenally

**Fig. 3:**
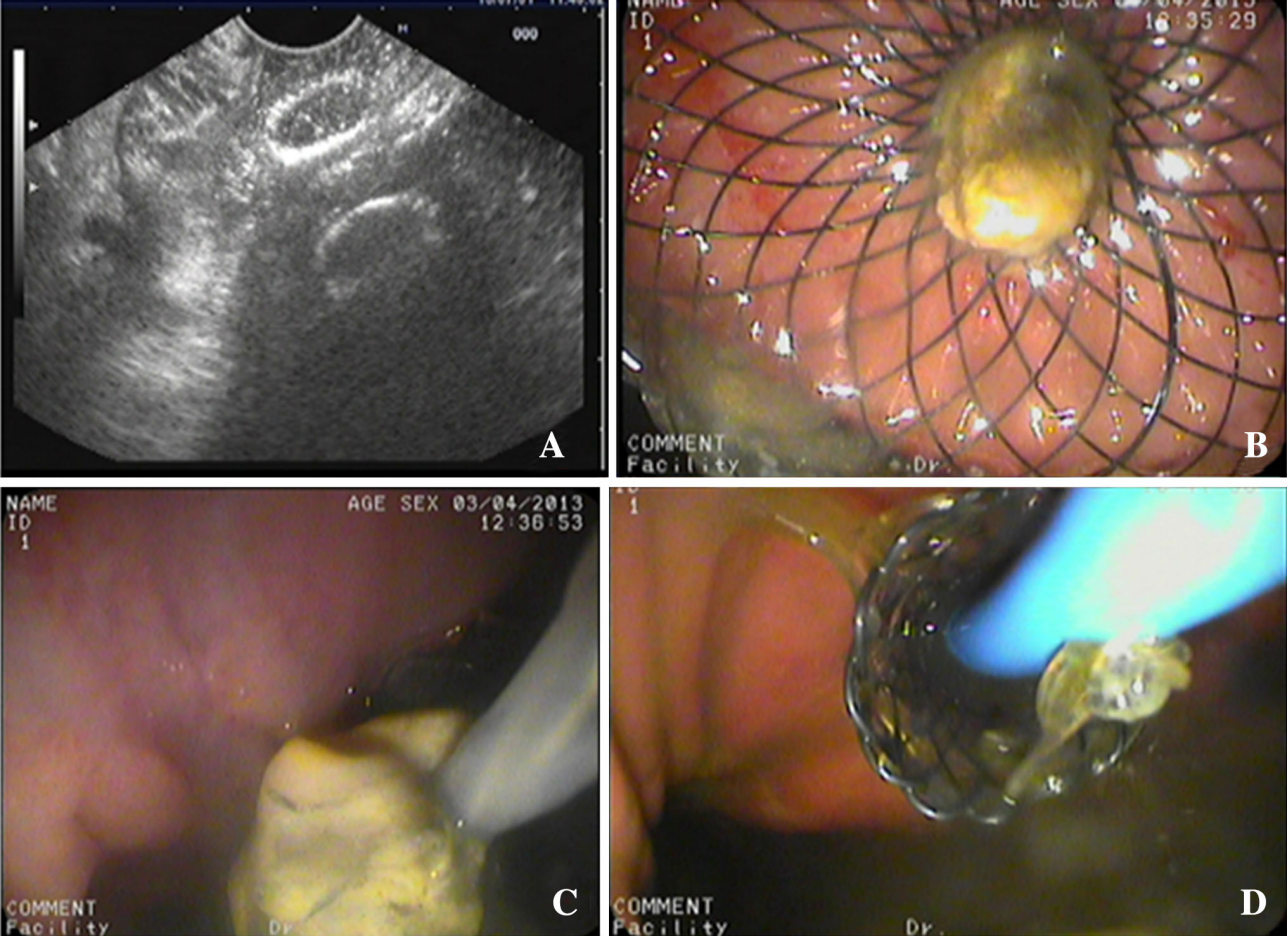
**A** In EUS imaging, a double-flanged metal stent was placed transmurally between the walled-off necrosis and gastric wall. **B**, **C** There was much debris in the cyst. Direct endoscopic necrosectomy was performed though the metal stent. **D** After necrosectomy, a pigtail-type, nasobiliary drainage catheter (7 Fr) was placed into the cyst

We enrolled 83 consecutive patients who underwent EUS-guided drainage for PFC at Shengjing Hospital of China Medical University from September 2008 to November 2012. Infections that occurred within 30 days after EUS-guided drainage were diagnosed by a physician according to the classical symptoms of fever, positive culture of aspirated fluid, and white blood cell elevation. The indications for EUS-guided drainage were: (1) symptomatic PFC; (2) PFC in which the cystic wall was in contact with the gastric or duodenal wall on EUS; and (3) PFC that was resistant to conservative treatment. Exclusion criteria were: (1) acute PFC; (2) acute necrotic collections (ANC); (3) non-fluid walled-off necrosis; and (4) patients with suspected malignancy (Figs. 1, 2, 3).

### Ethics

This study was approved by the Institutional Review Board and Ethics Committee of China Medical University. All patients voluntarily chose their therapeutic course and provided written informed consent for their participation in this study. Written informed consents were obtained from the parents or guardians of minors (age <18 years).

### Procedure

All procedures were conducted by an experienced therapeutic endoscopist. The instruments used were a linear array echo-endoscope (EG3830UT; Pentax, Tokyo, Japan) with an adjustable ultrasonic frequency of 5, 7.5, or 10 MHz, in combination with an ultrasound scanner (EUB 6500; Hitachi, Tokyo, Japan). The cysts were carefully observed to ensure that no mural nodules or mass lesions were being overlooked; then, the puncture site was determined. A prerequisite for needle placement was that the gastric or duodenal wall should be in contact with the cystic wall at the puncture site. Power Doppler imaging was used to confirm that there were no interposed vessels at the puncture site. The initial puncture was accomplished with a 19-gauge needle (EUS N-19-T; Wilson–Cook Medical, Winston–Salem, NC, USA) guided by real-time EUS imaging. After withdrawing the inside needle stylet, some fluid was aspirated for routine cytological, biochemical, and microbiological analysis as well as culture.

A 0.035-inch guidewire (Jagwire; Boston Scientific, Natick, MA, USA) was inserted through the needle lumen into the cyst. When fluoroscopic imaging confirmed that the guidewire was sufficiently inserted, the needle was withdrawn; the guidewire was left in situ. Subsequently, a cystotome (10 Fr; Wilson–Cook Medical) was used to dilate the tract and create a large fistula. After dilation of the puncture tract, a double-pigtail stent (8.5 or 10 Fr; Endo-Flex GmbH, Voerde, Germany) was placed into the cyst cavity over the guidewire. The guidewire was then removed, and the cystic fluid was aspirated via the drainage tube. Finally, the echo-endoscope was removed and the drain was fixed at an adequate position.

Post-procedure, the patients were observed for a period of at least 7 days. Prophylactic antibiotic (ceftriaxone, 1 g IV) was given routinely twice daily for at least 2 days after the procedure. After placement of the double-pigtail stent, the patients were followed up clinically and radiologically. Computed tomography (CT) was done 7 days post-procedure, and then once monthly. The double-pigtail stent was removed after the patients became asymptomatic for a period of at least 4 weeks, and after confirming the absence of a cyst cavity by CT. A pseudocyst was deemed to be resolved if the CT revealed no collection at 3 months post-procedure.

Post-procedure, if the body temperature of patient rose above 38.0 °C and persisted for >48 h, and the white blood cell count was >10^10^ mmol/L, an infection was assumed. Once infection occurred, dilation of the tract by wire-guided balloon up to 12–15 mm was conducted. After dilation of the puncture tract, another two or three double-pigtail stents (10 Fr; Endo-Flex GmbH) were placed in the cyst cavity. A pigtail-type nasobiliary drainage catheter (7 Fr; Wilson–Cook Medical) was sometimes placed into the cyst. Then, the cysts were routinely lavaged with normal saline. If there were much debris in the cyst, metal stents (10 mm diameter; Micro Technique, Nanjing, China) were used instead of double-pigtail stents. Direct endoscopic necrosectomy was performed though the metal stent. Conversely, if the cyst did not resolve or the symptoms persisted, alternative treatments such as percutaneous drainage or surgical intervention were considered.

### Statistical analysis

Separate multivariate logistic regression models for all EUS-guided drainage were carried out. For the logistic regression, the normality of residuals was tested by probability plots. Statistical analysis was performed with SPSS version 19.0 (SPSS Inc., Chicago, IL, USA). *P* < 0.05 was considered to be statistically significant.

## Results

From September 2008 to November 2012, 83 patients (45 male and 38 female, median age: 47.9 years, range 10–80 years) with PFCs were included. The baseline characteristics are presented in Table 1.

**Table 1:** Baseline characteristics of patients who underwent EUS-guided drainage

Total no. of patients	83
Age (year)	47.9
Male: female	45:38
Etiology	
Acute pancreatitis	40
Chronic pancreatitis	26
Trauma and surgery	14
Others	3
Location of the cyst	
Head	19
Body	24
Tail	40
Cyst diameter (cm)	11
Cyst wall thickness (mm)	5
Infected pseudocyst before EUS-guided drainage	8
Diabetes mellitus	12

The etiology of the PFCs was acute pancreatitis in 40 (48.2 %) patients, chronic pancreatitis in 26 (31.2 %), external injury and surgery in 14 (16.9 %), post-chemotherapy in one (1.2 %), and idiopathic in the remaining two (2.4 %). Sixty patients had a single cyst, and 23 had multiple cysts. The cysts were located in the pancreatic head in 19 patients, the body in 24, and the tail in the remaining 40 (only the cysts that underwent EUS-guided drainage were included). Eleven patients with cysts in the tail region had compartmental portal hypertension. The median largest diameter of the cysts was 11 cm (range 6–26 cm).

The puncture was attempted via the transgastric approach in 76 patients and via the transduodenal approach in seven. The median thickness of the cystic and gastric/duodenal walls was 5 mm (range 3–10 mm). EUS-guided drainage was successful in all patients. The clinical symptoms resolved in most patients after a median duration of 2 days (range 1–7 days). Seventeen patients had fever after EUS-guided drainage. The fever resolved within 48 h in ten patients following the administration of broad-spectrum antibiotics. A definite diagnosis of infection after EUS-guided drainage was made in seven other patients. All seven patients had a case history of severe acute pancreatitis, and all cyst diameters were >15 cm. Three patients had diabetes mellitus. Following dilation and stent change, the body temperature of all patients decreased to normal. No other treatment options were considered.

Univariate analysis of the risk factors for infection after EUS-guided drainage is presented in Table 2. In addition, multivariate analysis is presented in Table 3. Complete resolution of pseudocysts was documented in all 83 cases. The indwelling double-pigtail stent was removed in all cases after a median duration of 11 weeks (range 4–18 weeks). After median follow-up of 31 months (range 5–67 months), recurrence of PFC was observed in one patient. This patient had chronic pancreatitis, which was managed with another session of EUS-guided drainage. After median follow-up of 31 months, all patients who did not develop a cystic fluid infection improved without any clinical sequelae.

**Table 2:** Univariate analysis of the risk factors for infection after EUS-guided drainage

Variable	Infection after EUS-guided drainage	*P*
Age (year)		
<50	3/45 (6.7 %)	0.815
≥50	4/38 (10.5 %)	
Gender		
Male	3/45 (6.7 %)	0.815
Female	4/38 (10.5 %)	
Combined with diabetes mellitus		
Yes	3/12 (25.0 %)	0.095
No	4/71 (5.6 %)	
Etiology		
Acute pancreatitis	7/40 (17.5 %)	0.042^*^
Chronic pancreatitis	0/26	
Trauma and surgery	0/14	
Others	0/3	
Multiple cysts		
No	4/60 (6.7 %)	0.621
Yes	3/23 (13.0 %)	
Cyst location		
Head	0/19	0.303
Body	3/24 (12.5 %)	
Tail	4/40 (10.0 %)	
Cyst diameter (cm)		
<15 cm	0/66 (0 %)	<0.000*
≥15 cm	7/17 (41.1 %)	
Approach of EUS-guided drainage		
Transgastric	76/83 (91.6 %)	0.408
Transduodenal	7/83 (8.4 %)	
Stent diameter		
8.5 Fr		0.938
10 Fr

**Table 3:** Multivariate analysis of the risk factors for infection after EUS-guided drainage

Variable	*B*	SE	Wals	*df*	*P*	Exp (B)	Exp (B); 95 %CI
Etiology	0.615	0.584	1.11	1	0.292	1.85	0.589–5.815
Cyst diameter	−3.437	1.183	8.438	1	0.004*	0.032	0.003–0.327

*SE* standard error, *CI* confidence intervals for proportions

* Statistically significant

## Discussion

PFC can develop secondary to fluid leakage or liquefaction of pancreatic necrosis. PFCs are also seen in association with acute and chronic pancreatitis, abdominal trauma, and surgery [3–5]. EUS-guided drainage of PFC has become first-line therapy at many centers [3–6]. This is due to the ability of EUS to assess wall thickness, identify major vessels, and find the closest access to the fluid cavity [7–9]. The procedure creates an internal fistula; thus, it avoids the inconvenience of external drainage and the risk of cutaneous fistula formation. EUS-guided drainage has a technical success rate >90 % and a treatment success rate of 75–90 %, depending on the pseudocyst characteristics [10–13]. EUS-guided drainage is also less invasive than surgery and avoids adverse events related to percutaneous drainage [11, 14]. Furthermore, EUS can easily identify and distinguish the nature of the lesion, even if there is no noticeable bulge into the gastric lumen. In addition, the dynamic movements of the puncture needle during the procedure can be controlled and tracked longitudinally by the real-time image; thus, avoiding any inadvertent complications related to needle puncture. The additional advantage of color Doppler US is that it aids identification of the interposed vessels located along the course of the needle puncture, thus insuring their avoidance. Therefore, EUS-guided transmural puncture is significantly more reliable, dependable, and safer than the conventional techniques.

Infection is one of the complications after EUS-guided drainage, which may prolong the length of stay and increase the cost. For these reasons, it is important to establish the risk factors for infection after EUS-guided drainage. Our objective was to determine whether the risk of infection after EUS-guided drainage was associated with patient- and/or procedure-related factors.

Procedure-related risk factors for infection after EUS-guided drainage have been reported in several studies. A recent study by Puri et al. [15] reported that dilatation of a fistulous tract up to 15 mm in length and the simultaneous use of stents and a nasocystic tube, combined with normal saline irrigation, improved the outcome of the technique. Their outcome for cyst resolution was 87 %, which was superior to other studies [16–18]. Another study by Ali et al. [19] reported in patients with pseudocysts with viscous fluid containing solid debris that EUS-guided endoscopic drainage via a nasocystic drain alongside transmural stents resulted in a lower stent occlusion rate and better short-term clinical outcomes, compared with patients who underwent cyst drainage via transmural stents alone. Takao et al. [20] reported that transenteric drainage of pancreatic pseudocysts using a novel, lumen-apposing metal stent was accomplished with a high degree of technical and clinical success.

In the present study, after dilation of the puncture tract, an 8.5–10-Fr double-pigtail stent was placed into the cyst cavity. The procedure was successful in all patients. Neither the diameter of double-pigtail stent nor the approach of EUS-guided drainage (transgastric or transduodenal) significantly affected the incidence rate of infection (*P* = 0.938 and *P* = 0.408, respectively).

Acute pancreatitis was a relevant independent risk factor for infection after EUS-guided drainage (*P* < 0.05). This may have been due to some pancreatic fluid collection after acute necrotizing pancreatitis, containing necrotic tissue and debris, which could not be evacuated by a single plastics stent. Cyst diameter was also an independent risk factor for infection. Perhaps the increased risk of infection was because more time was needed for cyst shrinkage.

Several investigators have demonstrated that pseudocyst drainage complicated by infection can be improved by additional stent placement that enhances drainage [21]. Therefore, we placed another two or three double-pigtail stents (10 Fr in diameter and 5 cm in length) into the cysts. In two cases, double-flanged metal stents were used instead of double-pigtail stents. Direct endoscopic necrosectomy was performed through the metal stent by using the forceps and basket. After complete removal of all solid debris from the cavity, a pigtail-type, nasobiliary drainage catheter was placed into the cyst. Then, the cysts were routinely lavaged with normal saline. The body temperature of the patients returned to normal after the procedure. The use of conventional tubular self-expandable metal stents (SEMS) for pseudocyst drainage has been previously reported [20, 22–25]. Itoi et al. [20] used SEMS to resolve pseudocysts. All stents were successfully deployed without complications; the median time of placement was 35 days. There was no pseudocyst recurrence during median follow-up of 11.4 months. In the study of Talreja et al. [22], SEMS were used for drainage of PFC. Seventeen of 18 (95 %) patients responded successfully, with 14 (78 %) achieving complete resolution of their PFCs. In the study of Belle et al. [23], a special, self-expanding, partially covered, metal mesh stent was designed to keep the pancreaticogastrostomy open for drainage of walled-off necrosis and for further endoscopic necrosectomy. All the cases in that study had complete removal of necrotic masses without major complications. Leong et al. [24] reported a case with severe necrotizing gallstone pancreatitis complicated by infected pancreatic necrosis. Necrosectomy was performed to control the ongoing sepsis. Subsequently, there was a recurrence of an infected necrotic collection at the site of necrosectomy. Pancreatic duct stenting was performed to treat pancreatic duct leakage, followed by EUS-guided insertion of a fully covered SEMS to drain the infected fluid collection. There was rapid and complete clinical recovery. Wrobel et al. [25] used a new lumen-apposing metal stent to drain PFC and perform endoscopic necrosectomy. Success rate and PFC resolution were comparable to those with pigtail stents, with fewer complications. Seifert et al. [26] described the technique of endoscopic necrosectomy. After needle knife puncture and dilation from 8 to 20 mm, Seifert achieved direct endoscopic access to the pancreatic necrosis [26]. In our study, double-flanged metal stents were used instead of double-pigtail stents in two cases. Endoscopic necrosectomy was performed though the metal stent. The metal stents were removed by using biopsy forceps under EUS guidance 12 weeks after placement; no complications occurred.

## Conclusions

The cyst diameter was an independent risk factor for infection. Larger cysts with a diameter >15 cm should perhaps be drained initially with multiple pigtail or a larger diameter self-expandable metal stents to try to avoid infection. Further studies of endoscopic drainage of PFC are indicated to define terminology and develop meaningful comparisons.

## Electronic supplementary material

Below is the link to the electronic supplementary material.

Supplementary material 1 (WMV 241551 kb)
